# Synergistic enhancement of tenogenic differentiation in adipose-derived stem cells via TGF-β and laser therapy

**DOI:** 10.3389/fbioe.2026.1860498

**Published:** 2026-06-30

**Authors:** M. Mpanza, H. Abrahamse, A. Crous

**Affiliations:** Laser Research Centre, Faculty of Health Sciences, University of Johannesburg, Johannesburg, South Africa

**Keywords:** adipose-derived stem cells, photobiomodulation, regeneration, tenogenic differentiation, transforming growth factor-beta

## Abstract

Adipose-derived stem cells (ADSCs) are increasingly explored for tendon regeneration owing to their accessibility and multipotency. Low-level laser therapy (LLLT), also referred to as Photobiomodulation (PBM), has emerged as a promising non-invasive approach for modulating cellular behavior. However, directing their differentiation into tenocytes remains an ongoing focus of optimization. This study investigates the synergistic effects of transforming growth factor-beta (TGF-β) and PBM on the tenogenic differentiation of ADSCs. ADSCs were cultured and exposed to specific PBM wavelengths (525 nm, 825 nm, and consecutive irradiation) at 5 and 10 J/cm^2^, both in the presence and absence of TGF-β. Tenogenic differentiation was evaluated using morphological assessments, gene expression analysis of key tenogenic markers (SCX, TNC, COL1A1, COL3A1, and DCN), morphology changes, and biochemical assays. The results reveal that PBM, especially at 525 nm and 5 J/cm^2^, in conjunction with TGF-β, significantly enhances tenogenic gene expression, ECM protein deposition, and cellular alignment indicative of tenocyte phenotype. These findings support a promising dual-modality approach to enhance ADSC tenogenic commitment and inform future tendon tissue engineering strategies.

## Graphical Abstract

**Figure:**
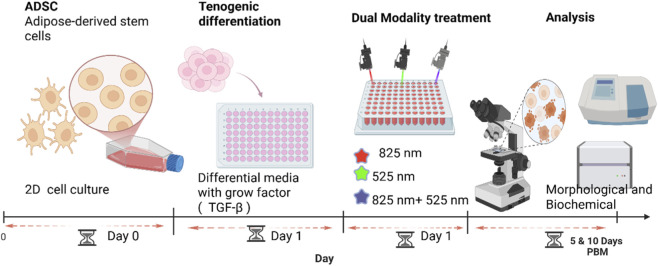

## Introduction

In regenerative medicine, optimizing stem cell differentiation strategies is essential, especially when developing successful treatments for musculoskeletal injuries. Among various stem cell sources, adipose-derived stem cells (ADSCs) have emerged as a promising candidate due to their relative abundance, minimally invasive harvest methods, high proliferative capacity, and multipotent differentiation potential (Zuk et al., 2001; Gimble et al., 2007). However, achieving efficient tenogenic differentiation remains complex and requires the coordinated use of biochemical cues to guide stem cell fate.

Biochemical factors, such as Transforming Growth Factor-beta (TGF-β), have emerged as potent teno-inductive agents. TGF-β promotes the expression of tenogenic markers such as scleraxis (Scx), tenomodulin, and collagen type I, all of which are essential for tendon formation and maturation (Shukunami et al., 2006; Chen et al., 2015). It also coordinates extracellular matrix remodeling, making it a cornerstone in tendon development and repair strategies (Liu et al., 2015).

Low-level laser therapy (LLLT), also referred to as Photobiomodulation (PBM), is a non-invasive light-based therapy that uses low-level lasers and has gained attention as an important tool to modulate cellular behavior. PBM exerts its effects by targeting cytochrome c oxidase in mitochondria, thereby enhancing adenosine triphosphate (ATP) production, reducing oxidative stress, and regulating critical signaling pathways involved in stem cell proliferation and differentiation (Farivar et al., 2014; Dompe et al., 2020). Notably, PBM has been shown to activate or modulate the Wnt/β-catenin pathway, a crucial pathway that intersects with TGF-β signaling to regulate stem cell fate and tenogenic gene expression (Bonnet et al., 2021; Jere and Houreld, 2022).

The combined use of PBM and TGF-β has shown promising results in promoting lineage-specific differentiation. However, optimizing this synergy depends heavily on PBM parameters such as wavelength, fluence, and irradiation duration, which influence tissue penetration depth, mitochondrial activation, and downstream gene expression (Hamblin, 2017; Da Silva et al., 2021). Of particular interest are the visible green wavelength (525 nm) and near infrared (NIR) wavelength (825 nm), which differ in tissue absorption and mitochondrial response. The 525 nm wavelength is absorbed more superficially and may influence early surface signaling events, while the 825 nm wavelength penetrates deeper and can stimulate mitochondrial-rich intracellular compartments (Crous et al., 2022; Fallahi et al., 2023).

Despite growing interest in dual-wavelength PBM, limited research has evaluated the combined effect of 525 nm and 825 nm delivered either individually or as consecutive irradiation at clinically relevant fluences of 5 J/cm^2^ and 10 J/cm^2^ on ADSC tenogenic differentiation, particularly within a TGF-enriched microenvironment. These fluences are bioactive and non-toxic in previous studies, making them suitable for investigating wavelength-specific and dose-dependent cellular responses (Dompe et al., 2020).

This study, therefore, aims to investigate the synergistic effect of TGF-β and PBM delivered at 525 nm, 825 nm, and consecutive 525/825 nm irradiation at fluences of 5 J/cm^2^ and 10 J/cm^2^ on the tenogenic differentiation of ADSCs. This approach holds significant promise for advancing tendon tissue engineering by identifying optimized, non-invasive strategies to enhance stem cell-based regenerative therapies.

We hypothesize that combining TGF-β with dual-wavelength PBM at 525 nm and 825 nm, particularly at optimized fluences of 5 J/cm^2^ and 10 J/cm^2^, will synergistically enhance tenogenic differentiation of ADSCs compared to single-wavelength irradiation or TGF-β treatment alone, through wavelength- and dose-dependent modulation of cellular signaling pathways involved in tendon lineage commitment.

## Materials and methods

### Experimental model

#### Cell culture

Immortalized ADMSCs (ASC52telo hTERT, ATCC® SCRC-4000™) were cultured in basal medium containing Dulbecco’s Modified Eagle Media (DMEM) (Sigma-Aldrich, D5796) supplemented with 10% foetal bovine serum (FBS Superior) (Biochrom, S0615) and 1% antibiotics: 0.5% Penicillin-Streptomycin (Sigma-Aldrich, P4333) and 0.5% Amphotericin B solution (Sigma-Aldrich, A2942). All cultured cells were maintained in Corning® cell culture flasks (Sigma, CLS430639/CLS430641/CLS431080) and incubated at 37 °C in 5% CO_2_ and 85% humidity (Heracell™ 150i CO2 Incubator, Thermo Scientific™, 51026280). The medium was refreshed every 2 days until the cells were 70%–80% confluent. Cells were then seeded into 96-well tissue culture-treated plates at 600 cells per well and allowed to attach for 24 h before irradiation.

#### Tenogenic differentiation

To induce tenogenic differentiation, ADMSCs were cultured in a custom-prepared tenogenic induction medium. The medium was composed of 125 µL of ascorbic acid (final concentration of 50 μg/mL), 250 µL of penicillin-streptomycin, 250 µL of amphotericin B, and 10 µL of TGF-β1, made up to a final volume of 25 mL using DMEM. All components were mixed under sterile conditions and used fresh to maintain the bioactivity of TGF-β1. A total of 600 ADMSCs per well were seeded into standard 96-well tissue culture plates, with each well containing 200 µL of the prepared induction medium. The cells were allowed to be attached for 24 h prior to PBM treatment. Although there is currently no universally accepted tenogenic differentiation protocol, the consecutive irradiation consecutive irradiation of TGF-β1 and ascorbic acid used in this study has demonstrated effectiveness in promoting the tenogenic commitment of ADMSCs, particularly when combined with PBM stimulation (Roets et al., 2025). Five cellular groups were established: Group 1 (no PBM or tenogenic media), Group 2 (tenogenic media only), Group 3 (825 nm, 5 J/cm^2^), Group 4 (525 nm, 5 J/cm^2^), and Group 5 (825 nm and 525 nm at 5 J/cm^2^ each).

#### Photobiomodulation for enhanced differentiation

Following seeding and cellular attachment, tenogenic differentiation media were replaced prior to irradiation. Cells were exposed to 825 nm diode laser (National Laser Centre of South Africa, SN 070900108), 525 nm diode laser (National Laser Centre of South Africa, EN 60825–1:2007), and consecutive dual-wavelength irradiation (525 nm followed sequentially by 825 nm) at fluences of 5 and 10 J/cm^2^.

For the dual-wavelength treatment, irradiation was delivered sequentially, with each wavelength applied independently. At the 5 J/cm^2^ condition, cells received 5 J/cm^2^ at 525 nm followed immediately by 5 J/cm^2^ at 825 nm. Similarly, at the 10 J/cm^2^ condition, cells received 10 J/cm^2^ at 525 nm followed immediately by 10 J/cm^2^ at 825 nm. This approach was used to evaluate the combined wavelength-specific effects of superficial green light stimulation and deeper near infrared penetration on ADSC tenogenic differentiation.

The power output (mW) of each laser was measured using a FieldMate Laser Power Meter and a High-Sensitivity Thermopile Sensor PM3 (Coherent, South Africa, 1,098,336). The laser parameters used in the present study are displayed in Table 1. Two post-irradiation experimental time points were selected for analysis: day 5 and day 10.

**TABLE 1:** Laser parameters.

Parameter	Green (G)	Near infrared (NIR)
Light source	Diode laser	Diode laser
Wavelength (nm)	525	825
Power output	548nW	129mW
Power density (mW/cm^2^)	10.22	53.53
Emission mode	Continuous wave	Continuous wave
Fluence (J/cm^2^)	5	5
Irradiation time	1 min 22 s (82 s)	5 min 20 s (320 s)

#### Giemsa and may-grünwald staining

On day 5 and day 10 post-seeding, cells were prepared for the Giemsa and May-Grünwald staining by removing the media from each well. The cells were then gently washed with 200 µL of 1X pre-warmed PBS to remove residual media. Fixation was carried out using absolute methanol, with 50 µL added per well, then incubated for 5 min at room temperature. Following fixation, each well received 50 µL of May-Grünwald stain, which was then incubated for 4 min to aid in the initial staining of cell components. 50 µL of Giemsa stain was added directly to the cells, followed by a 6 min incubation to enhance nuclear and cytoplasmic contrast (Piaton et al., 2015). After staining, cells were carefully washed three times with 200 µL of pre-warmed PBS, ensuring removal of excess dye. Finally, cells were imaged directly in the plate using a bright-field inverted light microscope (Olympus, CKX41), with the staining and consecutive irradiation enabling detailed visualization of cell morphology, including nuclear chromatin structure and cytoplasmic features.

#### Sirius red stain

Collagen was stained with 0.1% Sirius red (Sigma-Aldrich, 365548) in a picric acid solution. At days 5 and 10 post-PBM, cells were washed 3x with 200 µL of 1× pre-warmed PBS, fixed in 70% ethanol for 30 min, and washed again. Stained cultures were imaged directly using a bright-field inverted light microscope to assess extracellular collagen production. For cellular quantification, Sirius red stain was stabilized, and collagen production was quantified by solubilizing the bound dye with 50 µL of 0.1% NaOH in 0.1% absolute methanol, followed by a 30-min incubation at room temperature. The absorbance was then measured at 540 nm (Specifications, Product 2020).

#### Lactate dehydrogenase assay (LDH) (cytotoxicity)

The effects of PBM on ADMSC cell membrane integrity during tenogenic differentiation were quantitatively assessed by measuring the levels of cytosolic lactate dehydrogenase (LDH) released from ADMSCs with compromised membranes using the CytoTox 96® Non-Radioactive Cytotoxicity Assay (Promega, G179A). ADMSC culture medium was mixed with 50 µL of CytoTox 96® reagent in a 96-well plate, and the mixture was allowed to sit at room temperature in the dark for 10 minutes. Absorbance was then measured using the VICTOR Nivo® multimode plate reader (PerkinElmer, HH3) to quantify LDH release as an indicator of cell membrane damage.

#### Proliferation ATP

CellTiter-Glo® 3D ATP luminescence assay (Promega, G968A) was used to assess the cellular proliferation of ADMSC. In a 96-well opaque plate, 100 µL of ADMSC in media was combined with 100 µL of the CellTiter-Glo® 3D reagent. The cells were incubated for 30 min at room temperature in the dark after being mixed for 5 min on an orbital shaker. The VICTOR Nivo® multimode plate reader (PerkinElmer, HH35940080 EN) was used to measure colorimetric changes in relative light units (RLUs) after the incubation.

#### Quantitative polymerase chain reaction (qPCR) for tenogenic differentiation

qPCR was used to measure the expression levels of tenogenic and AMDSC marker genes in iADSC-differentiated ADMSC. Using the Quick-RNATM MiniPrep Plus Kit (Zymo, ZR R1058), total RNA was extracted from ADMSC. The RNA was then used with the LunaScript RT SuperMix Kit (ADMSC, E3010) to synthesize cDNA using universal reverse primers. The Agilent Aria MX Real-Time PCR (Agilent Technologies, G8830-64001) was used to amplify and quantify the target genes listed in Table 2 using the Luna Universal qPCR Master Mix (ADMSC, M3003). The conditions for all qPCR reactions were as follows: initial denaturation at 95 °C for 1 min, 40 cycles at 95 °C for 15 s, and annealing at 60 °C for 30 °C. The relative fold change was calculated using the 2^(−ΔΔCt)^ method, and the gene expression level was normalized to Glyceraldehyde 3-phosphate dehydrogenase (GAPDH).

**TABLE 2:** Primer sequences.

Primers	Forward primer (5′- 3′)	Reverse primer (5′ – 3′)
GAPDH	AGC​TGA​ACG​GGA​AGC​TCA​CT	TGC​TGT​AGC​CAA​ATT​CGT​TG
COL3A1	TGG​TCT​GCA​AGG​AAT​GCC​TGG​A	TCT​TTC​CCT​GGG​ACA​CCA​TCA​G
COL1A1	GAT​TCC​CTG​GAC​CTA​AAG​GTG​C	AGC​CTC​TCC​ATC​TTT​GCC​AGC​A
BGN	TGA​CTG​GCA​TCC​CCA​AAG​AC	GAG​TAG​CGA​AGC​AGG​TCC​TC
DCN	GCG​GAT​CCA​TCA​TGA​AGG​CAA​CTC	GCG​CTC​GAG​CTT​GTA​GTT​TCC​AAG​TT
TNC	GTC​ACT​CAT​CAC​AGC​TCT​GG	CTG​AGT​GTG​TAT​TCC​GTG​GC
MKX	ACG​AAG​ATC​TAT​GCG​GGA​A	GAG​CGT​CGA​CGG​GTT​TCA​GTC

#### Statistical analysis

Every vitro experiment was conducted three times (n = 3), and the experimental data are shown as means ± standard errors of the mean. To ascertain statistical significance, all *in vitro* data were statistically analyzed using GraphPad Prism (version 8.4) software. The statistical significance between the treatment groups and the control (untreated ADMSC) groups, as well as between the treatment groups, was assessed using the Turkey *post hoc* test and one-way analysis of variance (ANOVA) at a confidence interval of 0.95. Statistical significance was set at p < 0.05 and was also defined as *p < 0.05, **p < 0.01, ***p < 0.001, and ****p < 0.0001.

## Results

### Overview of the photobiomodulation (PBM) system in stem cell differentiation

To evaluate the capacity of Photobiomodulation (PBM) as a controllable, non-invasive regulator of stem cell fate, adipose-derived mesenchymal stem cells (ADMSCs) were subjected to defined visible (525 nm), near infrared (825 nm), and combined wavelength protocols under tenogenic induction conditions. The PBM system was assessed not only as an adjunct to biochemical differentiation cues, but as an active modulator of cellular architecture, extracellular matrix synthesis, metabolic activity, and lineage-specific gene expression. Outcomes were analyzed across two complementary experimental models: (i) a tenogenic differentiation model evaluating phenotypic and molecular progression over time, and (ii) a wavelength- and fluence-dependent PBM response model assessing how distinct photonic parameters influence stem cell behavior.

### PBM-induced morphological reorganization during tenogenic commitment

**FIGURE 1:**
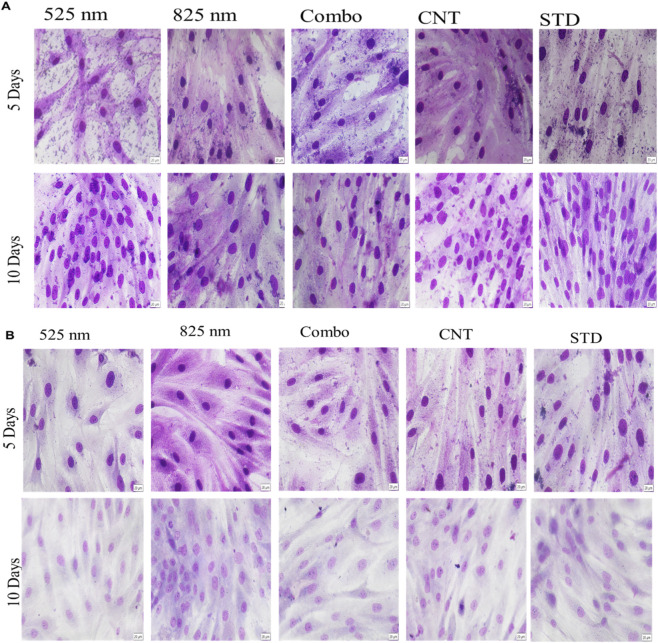
Giemsa-stained morphology of ADSCs undergoing tenogenic differentiation after PBM at 5 J/cm^2^ **(A)** and 10 J/cm^2^ **(B)** Representative Giemsa-stained micrographs of ADSCs at day 5 (top row) and day 10 (bottom row) following PBM treatment with 525 nm, 825 nm, and a consecutive irradiation of both wavelengths, in the presence of TGF-β. Control groups include CNT (TGF-β-enriched media without PBM) and STD (complete media without PBM or TGF-β). At day 5, early signs of elongation and alignment are visible, particularly in the 525 nm and consecutive irradiation groups at both fluences. By day 10, enhanced spindle-shaped morphology and nuclear alignment are prominent in all PBM-treated groups, particularly in the consecutive irradiation group, indicating progressive tenogenic differentiation. Scale bars = 20 µm.

Morphological analysis using May–Grünwald–Giemsa staining revealed progressive changes in cell shape, nuclear organization, and alignment consistent with tenogenic differentiation across all groups exposed to TGF-β–supplemented medium. However, PBM-treated groups exhibited earlier and more pronounced structural reorganization compared to non-irradiated controls. At day 5, for both fluences, cells exposed to 525 nm laser displayed moderate cell density with early signs of nuclear elongation and alignment, suggesting the onset of cytoskeletal reorganization consistent with early tenogenic differentiation. In contrast, cells treated with 825 nm showed a lower density and less morphological organization at this time point, indicating that the effects of this wavelength may require longer exposure to manifest. Notably, the consecutive irradiation treatment group (525 + 825 nm) exhibited the highest cell density and earliest onset of nuclear alignment, with cells appearing more compact and uniformly oriented, showing an indication of enhanced tenogenic commitment. By day 10, the differences became more pronounced at 525 nm, treated cells demonstrated increased elongation and clearer spindle-like morphology, while 825 nm treated cells caught up in density and alignment, now showing improved nuclear shape and orientation. The consecutive irradiation group continued to exhibit superior morphology, with tightly packed, elongated nuclei and highly organized alignment, reflecting synergistic effects of the two wavelengths on cellular architecture. In comparison, the Control (CNT) group (TGF alone) showed moderate improvements, with increased density and some cell alignment, but the morphological organization remained less uniform than in PBM-treated groups. The Standard (STD) group (complete media only) retained a more disorganized and rounded morphology across both time points, suggesting limited spontaneous differentiation. The consecutive irradiation of PBM with TGF resulted in the most pronounced histological features of tenogenic differentiation, supporting prior findings that PBM, especially when wavelengths are combined, can synergistically enhance stem cell morphology and lineage-specific differentiation (Figure 1). In B (10 J/cm^2^), the same time-dependent trend is observed, with Day-10 PB M groups displaying pronounced spindle morphology and nuclear alignment; the dual group appears most organized, with 525 nm close behind, while 825 nm and controls remain less oriented.

### PBM-mediated extracellular matrix formation and collagen deposition

**FIGURE 2:**
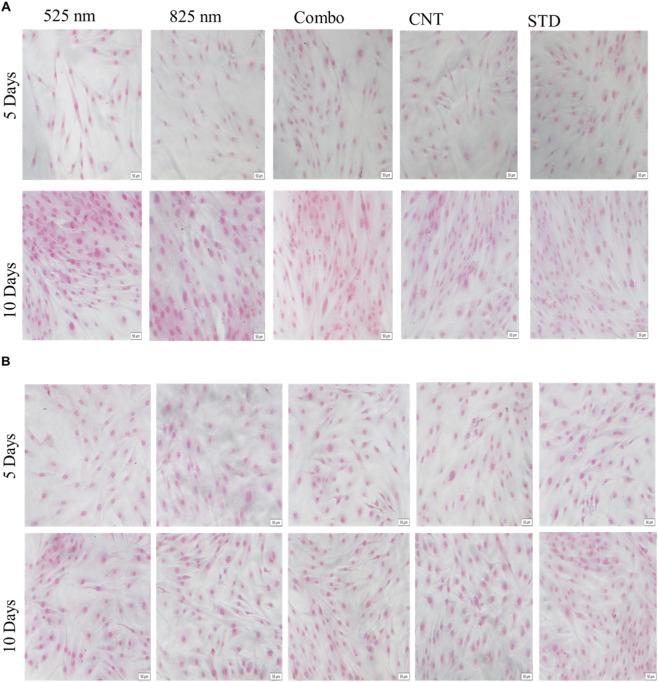
Sirius Red staining of collagen deposition in ADSCs after PBM at 5 and 10 J/cm^2^Post-PBM, Representative bright-field micrographs of extracellular collagen (Sirius Red) in ADSCs exposed to PBM at 525 nm, 825 nm, and 525 + 825 nm, delivered at **(A)** 5 J/cm^2^ and **(B)** 10 J/cm^2^. Top rows: Day 5; bottom rows: Day 10. All PBM groups were cultured in TGF-β–supplemented medium. TGF-β only (no PBM); Standard, complete medium only (no TGF-β, no PBM). Scale bars = 50 µm.

Sirius Red staining, which binds specifically to collagen, was performed to assess extracellular matrix (ECM) synthesis during tenogenic differentiation. On day 5, minimal collagen deposition was observed across all groups (Figure 2), with faint pink staining around the nuclei. This is consistent with the early stages of differentiation, where the onset of ECM production is just beginning. The consecutive irradiation group (525 + 825 nm) and the CNT (TGF only) group exhibited slightly more evident staining compared to others, suggesting early ECM activation influenced either by PBM or TGF.

By day 10, differences in collagen deposition became more evident in the 5 J/cm^2^. The 525 nm-treated group exhibited the most intense Sirius Red staining, with dense and widespread collagen fiber deposition around elongated nuclei. This morphological pattern strongly resembles aligned collagen fibrils associated with tenogenic matrix organization. The consecutive irradiation (525 + 825 nm) and 825 nm groups also showed collagen production, though less intense and uniformly distributed than 525 nm alone. The CNT group maintained moderate collagen staining, while the STD group showed some ECM deposition, potentially reflecting baseline matrix production by undifferentiated cells or early spontaneous differentiation.

### Quantitative interpretation of collagen

**FIGURE 3:**
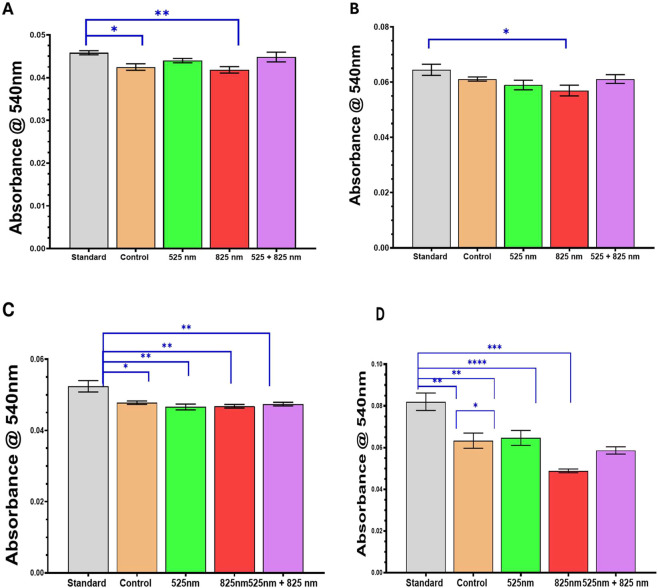
Quantification of collagen deposition in ADSCs following PBM treatment. **(A)** Day 5, 5 J/cm^2^; **(B)** Day 10, 5 J/cm^2^; **(C)** Day 5, 10 J/cm^2^; and **(D)** Day 10, 10 J/cm^2^. ADSCs were treated with 525 nm, 825 nm, and consecutive irradiation(525 + 825 nm) in the presence of TGF-β. Collagen levels were compared with the Control (TGF-β only) and Standard (complete medium only) groups. Significant differences are indicated as *p < 0.05, **p < 0.01, ***p < 0.001, and ****p < 0.0001. Data are presented as mean ± SEM (n = 3).

Across conditions, collagen deposition rose from day 5(A) to day 10(B). At 5 J/cm^2^, the consecutive irradiation group showed the greatest collagen on both days, with 525 nm next; the standard condition remained slightly above the control, while 825 nm was consistently low. At 10 J/cm^2^, the standard again ranked highest. On day 5(C), the control exceeded the consecutive irradiation, with 825 nm and 525 nm lower; but by day 10(D), 525 nm surpassed the control, the consecutive irradiation trailed the control, and 825 nm remained lowest as seen in Figure 3. Taking together, these patterns indicate a fluency-dependent shift in PBM effectiveness, an advantage for the consecutive irradiation of the 525 nm at the lower dose, and a 525 nm specific effect at the higher dose, while the 825 nm group underperformed across time points, consistent with NIR wavelengths modulating ECM synthesis differently from visible light.

### Metabolic activity and cytocompatibility during PBM-assisted differentiation

**FIGURE 4:**
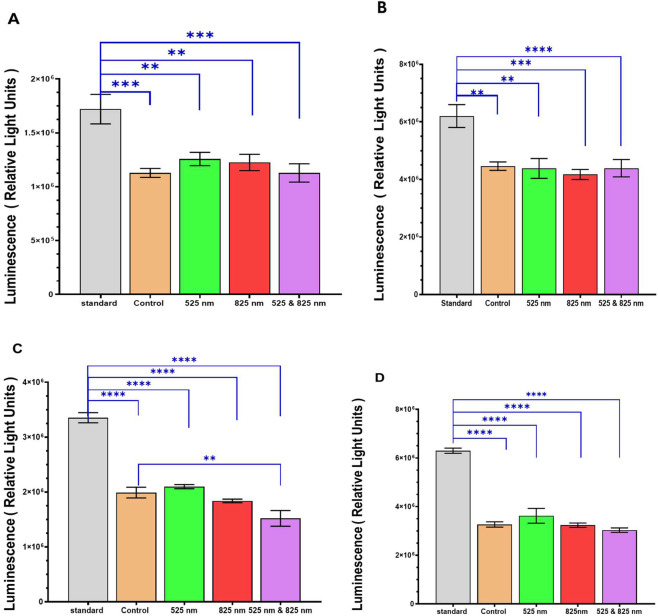
ATP-linked luminescence levels in ADSCs following PBM treatment. **(A)** Day 5, 5 J/cm^2^; **(B)** Day 10, 5 J/cm^2^; **(C)** Day 5, 10 J/cm^2^; and **(D)** Day 10, 10 J/cm^2^. ADSCs were exposed to 525 nm, 825 nm, or consecutive 525 + 825 nm irradiation in the presence of TGF-β. ATP levels were compared with the Control (TGF-β only) and Standard (complete medium only) groups. Significant differences are indicated as *p < 0.05, **p < 0.01, ***p < 0.001, and ****p < 0.0001. Data are presented as mean ± SEM (n = 3).

The metabolic profile of adipose-derived stem cells (ADSCs) undergoing tenogenic differentiation did not show a universal increase in activity but rather a dynamic shift in metabolic behavior as the cells transitioned into a tenocyte-like phenotype. These findings are not consistent with existing literature demonstrating that PBM within the 5–10 J/cm^2^ range promotes proliferation and mitochondrial activity in stem and fibroblast cells (Karu, 2010; AlGhamdi et al., 2012). This observation aligns with the concept that differentiating cells redirect metabolic resources from proliferation toward specialized functional pathways. Although no significant differences were noted between the Control and PBM-treated groups, the highly significant reductions in ATP relative to the Standard across both 5 and 10 J/cm^2^ fluences reflect that energy metabolism was dynamically modulated in response to the differentiation stimuli and PBM exposure (Figure 4). Interestingly, the strongest significance was observed in the consecutive irradiation (525 + 825 nm) group, suggesting that sequential wavelength exposure may induce a more pronounced metabolic reprogramming effect.

**FIGURE 5:**
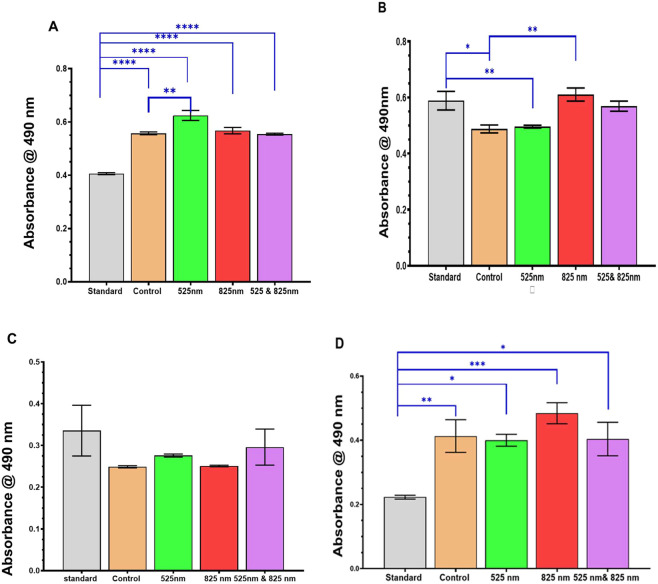
LDH cytotoxicity assessment in ADSCs following PBM treatment. **(A)** Day 5, 5 J/cm^2^; **(B)** Day 10, 5 J/cm^2^; **(C)** Day 5, 10 J/cm^2^; and **(D)** Day 10, 10 J/cm^2^. ADSCs were treated with 525 nm, 825 nm, or consecutive 525 + 825 nm irradiation in the presence of TGF-β. LDH release was used as an indicator of membrane integrity and cytotoxicity. Significant differences are indicated as *p < 0.05, **p < 0.01, ***p < 0.001, and ****p < 0.0001. Data are presented as mean ± SEM (n = 3).

The cytotoxicity assay was done to assess the membrane integrity of the cells, and the LDH levels remained low across all groups, with no significant increase observed in any of the PBM-treated cells for both day 5 and 10, as seen in Figure 5. The consecutive irradiation group maintained low LDH expression, further confirming that the reduced mitochondrial activity did not come at the cost of cellular stress or membrane damage. On day 5 at 5 J/cm^2^, 525 nm showed the highest LDH, followed by 825 nm, then control, with the consecutive irradiation lower and the standard lowest; at 10 J/cm^2^, the standard ranked highest, followed by the consecutive irradiation, then 525 nm, with 825 nm and control lowest and similar. On day 10 at 5 J/cm^2^, 825 nm was highest, followed by the standard, then the consecutive irradiation, with control at 10 J/cm^2^, 825 nm again ranked highest, followed by control, then the consecutive irradiation, with 525 nm lower and the standard lowest. Together, the ATP and LDH results indicate that PBM particularly stimulates mitochondrial function without inducing cytotoxicity, supporting a favorable environment for tenogenic differentiation when combined with TGF.

### Wavelength and fluence-dependent PBM regulation of tenogenic gene expression

Quantitative PCR analysis showed changes in tenocyte-associated gene expression following PBM across different wavelengths, fluences, and time points. Overall, gene expression levels tended to increase from day 5 to day 10 across most markers, suggesting a time-associated progression of tenogenic differentiation under both PBM and TGF-β culture conditions.

At 5 J/cm^2^, early markers such as SCX and COL3A1 showed modest increases, while later markers including TNC and COL1A1 demonstrated higher mean expression at day 10. At 10 J/cm^2^, a generally higher magnitude of gene expression was observed across several markers, particularly SCX, TNC, COL1A1, BGN, and DCN. The combined (525 + 825 nm) wavelength group frequently showed comparatively higher mean expression levels at day 10 across multiple genes, suggesting a possible additive or synergistic trend under dual-wavelength exposure.

However, despite these observable differences in mean expression patterns, two-way ANOVA analysis indicated no statistically significant effects of wavelength, fluence, time, or interactions (p > 0.05). Therefore, these variations should be interpreted as descriptive trends rather than statistically supported differences between treatment conditions.

**FIGURE 6:**
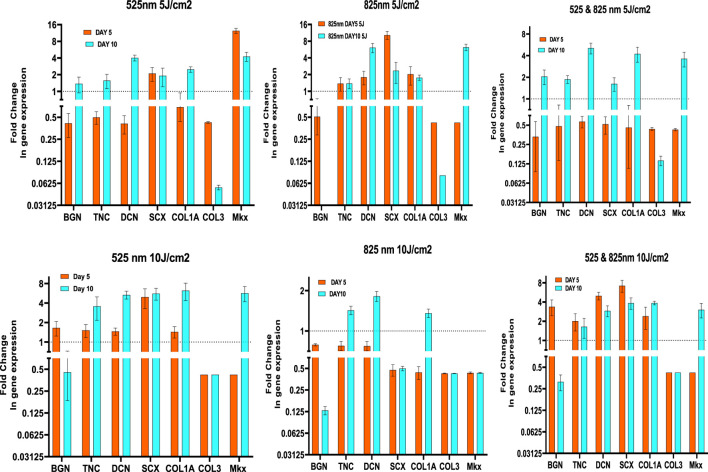
Quantitative PCR analysis of tenocyte-specific gene expression in ADMSCs following PBM treatment. Relative mRNA expression of SCX, TNMD, COL1A1, COL3A1, BGN, DCN, and TNC was assessed after 5 and 10 days of culture under 525 nm, 825 nm, and combined 525 + 825 nm irradiation at 5 J/cm^2^ and 10 J/cm^2^ and normalized to the untreated control (dashed line = 1). Overall, gene expression showed a general upward trend over time across all PBM conditions, with higher mean values frequently observed at day 10 and in the 10 J/cm^2^ and combined wavelength groups. Although early comparisons indicated upregulation of several tendon-related markers relative to untreated controls at day 5, subsequent two-way ANOVA restricted to PBM-treated groups revealed no statistically significant effects of wavelength, fluence, time, or interactions (p > 0.05). These findings, therefore, reflect descriptive trends rather than statistically confirmed differences between PBM conditions.

All expression data were normalized to housekeeping genes and validated for PCR efficiency. Results were presented on a logarithmic scale to facilitate visualization of relative gene expression changes (Figure 6).

These findings suggest a general tendency toward increased tenogenic gene expression over time under PBM conditions, but further studies with larger sample sizes and optimized experimental parameters are required to determine whether these trends represent reproducible treatment effects.

This observation aligns with the biphasic dose response (Arndt-Schulz curve) commonly reported in PBM studies, where low to moderate fluences stimulate cellular activity and gene expression, but higher fluences can inhibit gene expression due to photoinhibition or cellular stress response (Peplow et al., 2011; Rocha et al., 2022).

## Discussion

This study investigated the effect of PBM at 525 nm, 825 nm, and their consecutive irradiation at a 5 and 10 J/cm^2^ fluency in conjunction with TGF-β on the tenogenic differentiation of ADMSCs. Key cellular processes, including morphology, collagen deposition, mitochondrial activity, and cytotoxicity, were evaluated at 5- and 10-day post-irradiation to assess the effectiveness of these light treatments in promoting tenogenesis.

Morphological evaluation using Giemsa staining revealed that PBM, particularly the dual-wavelength treatment (525 + 825 nm), promoted aligned, elongated nuclei and increased cell density by day 10, with hallmarks of tenogenic transition. Similarly, the 525 nm group demonstrated clear spindle-shaped elongation consistent with cytoskeletal reorganization during tendon lineage commitment. These findings align with previous reports by (Crous et al., 2022; Da Silva et al., 2021), which emphasized the influence of PBM, especially when using multiple wavelengths, on enhancing stem cell alignment and orientation during differentiation. While the consecutive irradiation of 525 nm and 825 nm wavelengths improved morphological alignment, it did not outperform 525 nm alone in matrix synthesis or gene upregulation, suggesting potential wavelength-specific modulation or interaction effects that merit further investigation. The relatively lower effectiveness of 825 nm at the tested fluences aligns with reports that longer wavelengths may require different dosing or pulsing parameters to effectively stimulate ECM outcomes in tenogenic differentiation (De Freitas and Hamblin, 2016).

These findings are further supported by recent evidence demonstrating the importance of stem cell-based approaches in tendon regeneration. He et al. (2024) highlighted that tendon-derived stem cells (TDSCs) possess strong regenerative potential due to their ability to promote extracellular matrix remodeling, collagen synthesis, and tendon-specific differentiation within tissue-engineered environments. The authors further emphasized that growth factors and biophysical stimulation strategies are essential for optimizing tendon regeneration outcomes. This supports the present findings, where PBM combined with TGF-β enhanced cellular alignment, matrix organization, and tenogenic gene expression in ADMSCs. The improved morphological organization observed following PBM treatment, therefore, reinforces the concept that light-based biostimulation can serve as an effective adjunctive strategy in stem cell-mediated tendon tissue engineering.

Sirius Red staining was used to evaluate collagen deposition, which further supported these results. By day 10, staining intensity and areal coverage increased in all groups, with thicker pericellular fibrils and more continuous extracellular matrix sheets evident. Comparing wavelengths, collagen staining appeared most pronounced with 525 nm, followed by the dual 525 + 825 nm exposure, while 825 nm showed comparatively lighter signal; this pattern was appreciable at both 5 and 10 J/cm^2^, with the higher fluence highlighting contrasts between groups. Notably, the 525 nm group demonstrated the most intense collagen production by day 10, suggesting strong ECM remodeling. This observation corroborates the findings of De Freitas and Hamblin (2016), who showed that green light (525 nm) can stimulate collagen production through mitochondrial activation and ROS signaling. Interestingly, while the consecutive irradiation group yielded the highest morphological organization, it displayed slightly lower collagen output than 525 nm alone, possibly due to wavelength interference or differential transcriptional regulation, as also noted by (Amid et al., 2014). Similar findings have been reported by Seyyedin et al. (2024), who demonstrated that multi-wavelength PBM can enhance stem cell differentiation through complementary activation of cellular photoreceptors and intracellular signaling pathways. Importantly, the literature on PBM-guided tenogenesis of ADMSCs remains scarce compared with osteo-/chondrogenesis, but the results found indicate the following: (i) tenogenic media raise collagen output in ADMSCs, (ii) PBM enhances tendon repair quality and collagen alignment when used with stem cells *in vivo*, and (iii) optimal PBM windows are narrow and wavelength dependent. Our results extend this picture by showing that 525 nm alone or combined with the 825 nm can be used to boost collagen deposition during early tenogenic differentiation, whereas 825 nm may require different doses or schedules to avoid underperforming on ECM endpoints (Huang et al., 2009; Yang et al., 2013).

Recent bibliometric evidence further highlights the growing scientific interest in tendon-derived stem cell research and regenerative tendon engineering. Zhang et al. (2024) demonstrated that current global research hotspots in tendon stem cell biology increasingly focus on stem cell differentiation, extracellular matrix remodeling, tissue engineering scaffolds, and signaling pathways involved in tendon regeneration. The authors identified stem cell-based tendon repair combined with biological stimulation strategies as an emerging frontier in regenerative medicine. These trends strongly support the relevance of the current study, where PBM and TGF-β were used synergistically to enhance ADMSC tenogenic differentiation and extracellular matrix synthesis.

The ATP results were not consistent with the role of PBM in stimulating cytochrome c oxidase to promote ATP synthesis (Hamblin, 2017) because PBM-treated groups exhibited a consistent reduction in intracellular ATP levels relative to the Standard, indicative of increased ATP consumption associated with active-matrix production and lineage commitment. This observation aligns with the concept that differentiating cells redirect metabolic resources from proliferation toward specialized functional pathways. Although no significant differences were noted between the Control and PBM-treated groups, the highly significant reductions in ATP relative to the Standard across both 5 and 10 J/cm^2^ fluences reflect that energy metabolism was dynamically modulated in response to the differentiation stimuli and PBM exposure. Interestingly, the strongest significance was observed in the consecutive irradiation (525 + 825 nm) group, suggesting that sequential wavelength exposure may induce a more pronounced metabolic reprogramming effect. However, a plateau or slight reduction in ATP was noted in the consecutive irradiation group by day 10, potentially reflecting metabolic stabilization as cells reached a more differentiated, energy-efficient state, an effect also observed during neural differentiation in 3D cultures (Mulaudzi et al., 2024).

Cytotoxicity measured via LDH release remained low across all treatment groups, demonstrating that PBM at 5 J/cm^2^ is non-damaging and biocompatible within the tenogenic induction context. These results are in line with those of (Da Silva et al., 2021), who reported minimal membrane damage with comparable PBM parameters. Taken together with our ATP, LDH results indicate fluence- and wavelength-dependent PBM response during ADMSC tenogenic differentiation: lower fluence produced the most favorable energetic profile, while 525 nm repeatedly outperformed 825 nm on bioenergetics and cytotoxicity endpoints, and aligned with stronger matrix outcomes in our setting. This is consistent with the well-described biphasic (Arndt–Schulz) dose response in PBM, where low to moderate doses stimulate mitochondrial activity and downstream synthetic programs, whereas higher doses can become inhibitory depending on time and the wavelength (Huang et al., 2009; Hamblin, 2017).

The qPCR analysis assessed the expression of tenocyte-associated genes in ADMSCs following PBM treatment under varying wavelengths, fluences, and culture durations. Across all experimental conditions, gene expression exhibited a general tendency to increase from day 5 to day 10 for most markers evaluated, suggesting time-dependent progression toward a tenogenic-like phenotype. This observation is consistent with previous reports indicating that PBM may support sustained transcriptional activity through mitochondrial and redox-sensitive signaling pathways (Dompe et al., 2020; Zecha et al., 2016).

Recent studies further contextualize these findings within tendon regeneration biology. He et al. (2024) reported that tendon repair is driven by coordinated regulation of tendon-specific transcription factors, extracellular matrix deposition, and matrix remodeling during stem cell differentiation. In parallel, Zhang et al. (2024) highlighted that current tendon stem cell research is increasingly focused on molecular signaling networks and tissue engineering strategies, underscoring the relevance of biophysical stimulation approaches such as PBM in regenerative applications.

At 5 J/cm^2^, descriptive increases in early tenogenic markers (e.g., SCX and COL3A1) were observed in both 525 nm and 825 nm groups relative to baseline normalization. Similarly, the combined wavelength condition demonstrated comparatively higher mean expression levels for several genes at day 10. However, two-way ANOVA restricted to PBM-treated groups did not reveal statistically significant effects of wavelength, fluence, culture time, or their interactions (p > 0.05). Therefore, these observations should be interpreted as non-significant descriptive trends rather than evidence of treatment-dependent effects.

A comparable pattern was observed at 10 J/cm^2^, where higher mean expression values were frequently noted in the combined wavelength and later time-point groups, particularly for genes associated with extracellular matrix organization, including COL1A1, TNC, BGN, and DCN. While these patterns may suggest potential modulation of tenogenic-related gene expression under specific PBM conditions, the absence of statistically significant differences indicates that no definitive treatment effect can be established within the current dataset.

Expression patterns of ECM-associated proteoglycans (BGN and DCN) followed a similar trend, with modest increases observed at later points across treatment groups. These proteoglycans are recognized as critical regulators of collagen fibrillogenesis, extracellular matrix assembly, and growth factor sequestration during tendon development and repair (Nikoloudaki, 2022; Xu et al., 2022) but given the lack of statistically significant effects, these variations are best interpreted as biological variability or preliminary indications of potential responsiveness rather than confirmed PBM-induced regulation.

The entire qPCR data indicate that under the present experimental conditions, PBM does not produce statistically significant modulation of tenogenic gene expression in ADMSCs when combined with TGF-β. Nevertheless, the consistent time-associated increase across groups may reflect baseline differentiation dynamics driven primarily by culture conditions.

From a mechanistic perspective, these observations remain compatible with established models of PBM involving mitochondrial photostimulation, ROS-mediated signaling, and downstream modulation of pathways such as TGF-β and Wnt/β-catenin. These mechanisms are consistent with recent findings by Mgwenya et al. (2025), who reported that PBM regulates cellular responses through modulation of inflammatory and redox-sensitive signaling pathways, thereby influencing tissue repair and regenerative processes. However, given the absence of statistically significant effects, these mechanisms should be considered as theoretical frameworks rather than directly supported drivers of the observed gene expression patterns in this dataset.

Further studies with increased sample sizes, refined dosing regimens, and enhanced experimental resolution are required to determine whether the observed trends reflect reproducible biological effects of PBM on tenogenic differentiation.

Recent evidence from He et al. (2024) further demonstrated that stem cell-mediated tendon regeneration is closely associated with the activation of signaling pathways that regulate tendon lineage commitment and extracellular matrix formation. Their review emphasized the role of tendon-specific transcription factors, collagen synthesis, and matrix remodeling during stem cell differentiation, supporting the present observations of increased SCX, COL1A1, TNC, BGN, and DCN expression following PBM treatment. Furthermore, Zhang et al. (2024) identified molecular signaling pathways and stem cell differentiation mechanisms as major emerging research hotspots in tendon regeneration studies, highlighting the increasing focus on optimizing regenerative signaling strategies such as PBM and growth factor stimulation.

Taken together, these findings reinforce the potential of PBM, particularly at 525 nm, alone or combined with 825 nm, as a powerful, non-invasive assistant to conventional growth factor-based tenogenic induction protocols. PBM modulates multiple cellular mechanisms, including cell morphology, mitochondrial function, and transcriptional activity, thereby enhancing ADSC differentiation toward tenocytes while preserving cell viability. Therefore, precise optimization of PBM parameters such as wavelength, fluence, and dose frequency is essential to maximize therapeutic efficacy by balancing ECM production with cellular health and lineage fidelity (Mulaudzi et al., 2024).

In agreement with this, He et al. (2024) emphasized that successful tendon tissue engineering requires the integration of stem cells, biological signaling molecules, and supportive biophysical stimulation to recreate the native tendon microenvironment and improve regenerative outcomes.

Furthermore, the mechanistic effects observed in this study can be further explained by the established role of PBM in mitochondrial photoreception and downstream signaling cascades. PBM primarily targets mitochondrial chromophores, particularly cytochrome c oxidase, leading to enhanced electron transport chain activity, transient increases in mitochondrial membrane potential, and the controlled production of reactive oxygen species (ROS). These ROS, at non-cytotoxic levels, function as critical secondary messengers that activate redox-sensitive signaling pathways. One such pathway includes the modulation of transforming growth factor-beta (TGF-β) signaling, a key regulator of tenogenic differentiation. ROS-mediated activation can enhance TGF-β ligand availability and receptor sensitivity, thereby promoting the phosphorylation of SMAD2 and SMAD3 proteins. These receptor-regulated SMADs subsequently form complexes with SMAD4, translocate to the nucleus, and regulate the transcription of tenogenic genes such as SCX, COL1A1, and TNMD. This PBM-induced ROS–TGF-β–SMAD axis provides a mechanistic link between mitochondrial activation and the transcriptional upregulation observed in this study. In addition, recent evidence suggests that PBM-mediated ROS signaling may also intersect with Wnt/β-catenin pathways to further regulate stem cell fate decisions and tenogenic differentiation, highlighting the complex signaling crosstalk involved in PBM-enhanced tendon regeneration.

These observations are further supported by recent tendon stem cell research trends identified by Zhang et al. (2024), where molecular signaling regulation, extracellular matrix remodeling, and tissue engineering integration were identified as key future directions in regenerative tendon medicine. Collectively, recent studies have highlighted the importance of PBM-mediated signaling, extracellular matrix regulation, and stem cell differentiation in regenerative medicine (Mgwenya et al., 2025; Nikoloudaki, 2022; Seyyedin et al., 2024; Xu et al., 2022).

This signaling interplay supports extracellular matrix synthesis and cytoskeletal organization, aligning with the enhanced collagen deposition and morphological changes reported. Thus, PBM not only influences cellular metabolism but also orchestrates key differentiation pathways through tightly regulated redox and growth factor signaling networks, strengthening its translational relevance for tendon tissue engineering and regenerative medicine applications.

## Conclusion and future directions

This study demonstrates that Photobiomodulation (PBM), particularly at 525 nm and in consecutive irradiation with TGF-β, promotes the tenogenic differentiation of ADMSCs without inducing cytotoxicity. PBM enhanced key differentiation markers at the morphological, biochemical, and genetic levels, including increased cell elongation and alignment, elevated collagen deposition, ATP production, and upregulation of tenogenic genes such as MKX, BGN, DCN, and TNC. The consecutive irradiation of 525 and 825 nm wavelengths showed descriptive synergistic trends in cell morphology and viability, while 525 nm alone appeared most effective for extracellular matrix remodeling.

The collagen deposition results echo prior work indicating that ADMSCs can be guided to become tendon-like cells capable of producing collagen-rich extracellular matrix under appropriate biochemical and biophysical cues. In addition, PBM has been widely reported to support collagen organizations during tendon repair, provided that wavelength and fluence remain within a narrow therapeutic window. The present findings further support PBM as a potentially useful adjunct to growth factor-based tenogenic induction strategies.

A key limitation of this study is the relatively small sample size (n = 3), which may limit statistical power to detect subtle differences between PBM conditions. In addition, the absence of statistically significant differences in gene expression suggests that some of the observed effects may represent biological trends rather than robust treatment-driven responses under the current experimental design.

Future experiments could refine the dose–response relationship around 525 nm (2–8 J/cm^2^), explore pulsing regimens and timing of TGF-β administration, and assess whether modified 825 nm parameters may better support extracellular matrix accumulation rather than predominantly proliferative responses (Huang et al., 2009; Yang et al., 2013). Expanding the experimental design to include larger cohorts, additional time points, and protein-level validation (e.g., Western blotting or immunostaining) may further strengthen the mechanistic interpretation of PBM effects.

These findings highlight PBM as a non-invasive, safe, and tunable tool for supporting tendon regeneration strategies and provide a foundation for further optimization of light-based protocols in musculoskeletal tissue engineering. However, given the absence of statistically significant differences in gene expression between PBM conditions, these molecular observations should be interpreted as preliminary biological trends rather than definitive treatment effects. Further studies should investigate long-term outcomes, earlier molecular responses, and potential combinatory effects with other bioactive cues to fully harness PBM’s regenerative potential.

## Data Availability

The original contributions presented in this study are included in the article and its Supplementary Material. The datasets generated and analyzed during the current study, including microscopy images and quantitative assay data, are available from the corresponding author upon reasonable request. Further inquiries can be directed to the corresponding author.
